# A Novel Vascular Endothelial Growth Factor Receptor Participates in White Spot Syndrome Virus Infection in *Litopenaeus vannamei*

**DOI:** 10.3389/fimmu.2017.01457

**Published:** 2017-11-01

**Authors:** Shihao Li, Zhiwei Wang, Fuhua Li, Kuijie Yu, Jianhai Xiang

**Affiliations:** ^1^Key Laboratory of Experimental Marine Biology, Institute of Oceanology, Chinese Academy of Sciences, Qingdao, China; ^2^Laboratory for Marine Biology and Biotechnology, Qingdao National Laboratory for Marine Science and Technology, Qingdao, China; ^3^University of Chinese Academy of Sciences, Beijing, China

**Keywords:** VEGFR, white spot syndrome virus, immune response, protein interaction, signaling pathway

## Abstract

Vascular endothelial growth factor (VEGF) signaling pathway is known to play key roles in endothelial cell proliferation, migration, angiogenesis, vascular permeability, inhibition of apoptosis, and virus infection. In the present study, a novel VEGFR gene (*LvVEGFR2*) was identified and characterized from *Litopenaeus vannamei*. The deduced amino acid sequence of *LvVEGFR2* possessed typical features of VEGFRs reported in other species, including six IG-like domains, a transmembrane motif, a protein kinase (PK) domain, and one tyrosine-PK active site. The transcripts of *LvVEGFR2* were mainly detected in hemocytes and lymphoid organ (Oka). Subcellular localization analysis showed that LvVEGFR2 was a membrane protein. Its expression level was obviously upregulated in hemocytes and Oka of the shrimp after white spot syndrome virus (WSSV) infection. Knockdown of *LvVEGFR2* gene expression by double-strand RNA mediated interference could lead to a decrease of virus copy number in WSSV-infected shrimp. The interaction between LvVEGFR2 and different LvVEGFs (LvVEGF1, LvVEGF2, and LvVEGF3) in shrimp was analyzed at the transcription level and protein level, respectively. Knockdown of *LvVEGF2* or *LvVEGF3* could downregulate the expression level of *LvVEGFR2*, and injection of the recombinant LvVEGF2 or LvVEGF3 could upregulate the expression level of *LvVEGFR2*. Yeast two-hybrid analysis showed that LvVEGFR2 could interact with LvVEGF2 and LvVEGF3 directly. The study improved our understanding on the VEGF signaling pathway of shrimp and its role during WSSV infection.

## Introduction

The vascular endothelial growth factor (VEGF) signaling pathway plays crucial roles in promoting cell proliferation, cell migration, increasing the vasopermeability, and promoting angiogenesis (1). VEGFs and their receptors, VEGFRs, are primary members of the VEGF signaling pathway. In mammals, there are five VEGF genes including VEGF-A, VEGF-B, placental growth factor, VEGF-C, and VEGF-D, and three VEGFRs including VEGFR1, VEGFR2, and VEGFR3 (2). The VEGF signaling pathway could be activated through binding of VEGF ligands to their receptors. The second and third extracellular immunoglobulin (Ig)-like domains of VEGFRs are necessary for high-affinity binding of VEGF ligands (3, 4). Different VEGFRs play various functions through binding to different VEGF ligands. VEGFR1 plays roles in recruitment of hematopoietic stem cells, migration of monocytes and macrophages as well as in cellular energy metabolism by binding to VEGF-A and VEGF-B (5, 6). VEGFR2 could regulate lymphatic endothelial cells and vascular endothelial cells, promote lymphatic and vascular production, as well as regulate lymphocyte migration after activation by VEGF-A and VEGF-D (7). VEGFR3 was considered to be involved in lymphangiogenesis and maintenance of the lymphatic endothelium by interacting with VEGF-C and VEGF-D (8, 9).

Vascular endothelial growth factor signaling pathway is also involved in the interaction between pathogens and host cells. The Parapoxvirus *Bovine papular stomatitis virus* and *Orf virus* could encode VEGF homologs which bind to VEGFR2 of the host cells and then participate in the process of viral infection (10–12). In crustacean, several VEGF genes have been identified and reported to be involved in the host-pathogen interaction. In *Eriocheir sinensis*, the VEGF homologous gene *EsPVF1* could be activated by *Vibrio anguillarum* and *Pichia pastoris* GS115 (13). In *Marsupenaeus japonicus*, two types of VEGF, *MjVEGF* and *MjVEGF2*, were also involved in innate immunity (14). In our previous study, we found that the transcription levels of the genes in VEGF signaling pathway were obviously upregulated in shrimp *Fenneropenaeus chinensis* under acute infection of white spot syndrome virus (WSSV) (15). Subsequently, we isolated three VEGF genes (*LvVEGF1, LvVEGF2*, and *LvVEGF3*) and a receptor gene *LvVEGFR* from *L. vannamei*. Silencing of these genes by dsRNA-mediated RNAi could reduce the *in vivo* copy number of WSSV and the accumulative mortality rate of the infected shrimp decreased apparently (16–18). These data suggested that the VEGF and VEGFR genes played important roles in the host immunity. However, the knowledge about the VEGF signaling pathway in crustacean is still very limited.

In the present study, a novel VEGFR gene, *LvVEGFR2*, from the whiteleg shrimp *L. vannamei* was identified. The tissue distribution of *LvVEGFR2* and its response to WSSV infection were analyzed. Double-strand RNA mediated RNA interference was performed to study the function of *LvVEGFR2* gene during WSSV infection. Furthermore, the study also analyzed the interaction between LvVEGFR2 and each LvVEGF. The data will not only clarify the function of *LvVEGFR2* gene during WSSV infection but also improve our understanding on the VEGF signaling pathway in the crustacean.

## Materials and Methods

### Experimental Animals

Healthy adult Pacific whiteleg shrimp cultured in our lab, with a body length of 16.1 ± 2.0 cm and a body weight of 12.1 ± 1.30 g, were used for tissue distribution analysis. Shrimp with a body length of 7.6 ± 0.5 cm and a body weight of 3.5 ± 1.0 g were used for WSSV infection, RNA interference, and recombinant protein injection experiments. All shrimp were acclimated in air-pumped circulating sea water at 25°C before experiment.

### Tissue Collection

For tissue distribution analysis, hemolymph of 12 healthy adult shrimp were obtained by using a sterile syringe preloaded with equal volume of modified anticoagulant Alsever solution and then centrifuged immediately at 800 g at 4°C for 10 min (19). The hemocytes were collected and preserved in liquid nitrogen. Then, the hepatopancreas, heart, muscle, lymphoid organ (Oka), intestine, stomach, gill, epidermis, eyestalk, testis, and ovary were dissected and kept in liquid nitrogen.

For WSSV challenge experiment, virus particles were prepared according to the method described by Sun et al. (20). The extracted WSSV was diluted to 500 copies/μl with sterile phosphate-buffered saline (PBS). Experimental animals were divided into 2 groups including WSSV group and PBS group, with 64 individuals in each group. 20 µl WSSV solution containing 10^4^ copies of WSSV was injected into each shrimp in the WSSV group and an equal volume of PBS was injected into each shrimp in the PBS group. All shrimp were cultured in air-pumped circulating sea water with a temperature of 25 ± 1°C. Hemocytes and lymphoid organ (Oka) of 12 shrimp in each group were collected separately at 1, 3, 6, 12, and 24 hour (h) post WSSV infection (hpi).

### Total RNA Extraction and cDNA Synthesis

Total RNA of each tissue was extracted using RNAiso Plus reagent (Takara, Beijing) following the manufacturer’s protocol. The RNA concentration was assessed by Nanodrop 2000 (Thermo Fisher Scientific, USA) and the RNA quality was assessed by electrophoresis on 1% agarose gel. All cDNA samples were synthesized from 1 µg total RNA with PrimeScript RT Reagent Kit (Takara, Beijing). First, genomic DNA (gDNA) was removed with gDNA Eraser. And then, the first-strand cDNA was synthesized by PrimeScript RT Enzyme following the manufacturer’s instructions with random primers.

### Cloning of the Open Reading Frame (ORF) Sequence of LvVEGFR2

The ORF sequence of *LvVEGFR2* cDNA was obtained from an Illumina-based transcriptome sequencing database of *L. vannamei* built in our lab (21). Primers LvVEGFR2-F1/LvVEGFR2-R1 and LvVEGFR2-F2/LvVEGFR2-R2 (Table 1) were designed to validate the sequence. The PCR program for the amplification of *LvVEGFR2* was as follows: 1 cycle of denaturation at 94°C for 5 min, 35 cycles of denaturation at 94°C for 45 s, annealing at 57°C for 45 s, and extension at 72°C for 45 s, followed by an extension at 72°C for 7 min. The specific products were assessed by electrophoresis on 1% agarose gel. Then, the amplified products were purified using TIANgel Midi purification kit (Tiangen, China) and cloned into pMD19-T vector (Takara, Beijing) and then transformed into Trans 5α competent cell for sequencing.

**Table 1:** Information of primer sequences used in the present study.

Primer name	Primer sequence (5′-3′)	Expected size (bp)	Annealing temperature (°C)	Primer sites (bp)
LvVEGFR2-F1	GCAAGGTATTTATCCCAGTT	1,956	56	145
LvVEGFR2-R1	CTTTAGGGTTAGGTTCTCAT			2,100
LvVEGFR2-F2	CGATTATGAGAACCTAACCCT	1,800	57	2,076
LvVEGFR2-R2	TCCTGAAGCCACAGCGATT			3,875
18S-F	TATACGCTAGTGGAGCTGGAA	136	56	–
18S-R	GGGGAGGTAGTGACGAAAAAT			–
LvVEGFR2-qF	TGCTCCTCGCAGACAACAAT	188	57	3,293
LvVEGFR2-qR	CCACAGAGTCACTCCAAAGG			3,480
VP28-qF	AAACCTCCGCATTCCTGTGA	141	55	–
VP28-qR	TCCGCATCTTCTTCCTTCAT			–
LvVEGFR2-dsF	TAATACGACTCACTATAGGG	701	59	2,739
	GGCTTACTTGGGCAAACAT			
LvVEGFR2-dsR	TAATACGACTCACTATAGGG			3,439
	TGAAGAAACGGTCACGAAT			
dsEGFP-F	TAATACGACTCACTATAGGG	289	59	–
	CAGTGCTTCAGCCGCTACCC			
dsEGFP-R	TAATACGACTCACTATAGGG			–
	AGTTCACCTTGATGCCGTTCTT			
LvVEGF1-pF	CCAAGATCTCTTTAACCCGATCTTCAGGG	848	55	–
LvVEGF1-pR	ACCAAGCTTTCAGAATAGCAAGGGTACAC			–
LvVEGF2-pF	CCAAGATCTGACCTTCGTTTATCCTGCCG	931	58	–
LvVEGF2-pR	ACCAAGCTTTTACGCGGGGCTACCTTCTG			–
LvVEGF3-pF	CCAAGATCTGAGGGGGTGCTTAAGCATTC	650	55	–
LvVEGF3-pR	ACCAAGCTTTCAGTCCATGCACCTGCATG			–
LvVEGFR2-pF	TTCAACCCAGTGCATGTTGAGGAG	2,061	55	817
LvVEGFR2-pR	CACAACTACTAGCCTCAGTTTGCG			2,289
LvFAK-qF	ATTACTCAACACCAGCAACC	172	57	–
LvFAK-qR	GTTCCCTCGGACTCCACCTT			–
LvPI3K-qF	TATGAAGTAACCCGTAGTGCCA	187	57	–
LvPI3K-qR	TGCCCACATCTCCTGACTGA			–

### Sequence Analysis

The complete ORF region and amino acid sequence of *LvVEGFR2* was deduced using ORF finder.^1^ The ORF sequence and deduced protein sequence of LvVEGFR2 were analyzed with BLAST algorithm at the National Center for Biotechnology Information^2^ and InterProScan software^3^ and Simple Modular Architecture Research Tool.^4^ Eight protein sequences of VEGFR family members from different species were used to perform multiple alignments using ClustalW2^5^ and a phylogenic tree was constructed by the neighbor-joining (NJ) algorithm using the MEGA 4 software. The reliability of the tree was tested by bootstrapping using 1,000 replications. The information of VEGFRs used in the present study was shown in Table 2.

**Table 2:** Information of VEGFR family members used for phylogeny analysis.

Gene symbol	Annotation	Accession number	Species
LvVEGFR1	Vascular endothelial growth factor (VEGF) receptor	KM280384	*Litopenaeus vannamei*
LvVEGFR2	VEGF receptor 2	MF417824	*L. vannamei*
DmPVR	PDGF- and VEGF-receptor related, isoform H	NP_001162909	*Drosophila melanogaster*
MdVEGFR1	VEGF receptor 1-like isoform X5	XP_005179579	*Musca domestica*
HsVEGFR1	VEGF receptor 1 isoform 1 precursor	NP_002010	*Homo sapiens*
HsVEGFR2	VEGF receptor 2 precursor	NP_002244	*H. sapiens*
HsVEGFR3	VEGF receptor 3 isoform 1 precursor	NP_891555	*H. sapiens*
BiVEGFR1	VEGF receptor 1	XP_012245575	*Bombus impatiens*
ZnVEGFR2	VEGF receptor 2	XP_021928982	*Zootermopsis nevadensis*
AtcVEGFR1	VEGF receptor 1	KYM91172	*Atta colombica*
ApcVEGFR	VEGF receptor	PBC30758	*Apis cerana*
LsVEGFR2	VEGF receptor 2	OWK64571	*Lonchura striata domestica*
CcVEGFR	VEGF receptor	CAA58267	*Coturnix coturnix*
DrVEGFR1	VEGF receptor 1	NP_001014829	*Danio rerio*
DrVEGFR2	VEGF receptor 2	NP_001019824	*D. rerio*
DrVEGFR3	VEGF receptor 3	XP_009306067	*D. rerio*

### Quantitative Real-time PCR Detection of LvVEGFR2 mRNA Expression

SYBR Green-based quantitative real-time PCR (qPCR) was performed to detect the gene expression level of *LvVEGFR2*. A pair of primers LvVEGFR2-qF and LvVEGFR2-qR (Table 1) was designed to detect the expression level of *LvVEGFR2* in different tissues and samples after WSSV infection. Primers 18S-F and 18S-R (Table 1) were designed to detect the expression of the internal reference gene, 18S rRNA. The program was as follows: denaturation at 94°C for 2 min; 40 cycles of 94°C for 15 s, annealing temperature for 20 s, and 72°C for 20 s. The PCR product was denatured to produce melting curve to check the specificity of the PCR product.

### Construction of pEGFP-N1 Plasmid with the Transmembrane Motif of LvVEGFR2 and Transfection into the Mammalian 293T Cells

A plasmid containing the predicted transmembrane motif (TM) of LvVEGFR2 was constructed to study the subcellular localization of LvVEGFR2 protein in mammalian 293T cells. The plasmid, designated as pEGFP-VR2, was constructed based on the expression plasmid pEGFP-N1 (Clontech, USA). The nucleotide sequence between the restriction sites *Age* I and *Not* I on pEGFP-N1 was replaced by a designed nucleotide sequence which encoded a IgK_secretion tag (METDTLLLWVLLLWVPGSTGD), the full length of enhanced green fluorescent protein (EGFP), and the predicted TM of LvVEGFR2 and its flanking sequence (from Val^644^ to Ser^702^), and flanked with the restriction sites *Age* I and *Not* I. The designed nucleotide sequence was synthesized by Sangon Biotech company (Shanghai, China) and sub-cloned into pEGFP-N1 using the restriction sites *Age* I and *Not* I. Three micrograms of the plasmids pEGFP-VR2 and pEGFP-N1 were transfected into the mammalian 293T cells with Lipofectamine 3000 Reagent (Thermo Fisher Scientific, USA) following the manufacture’s instruction, respectively. The transfected cells were cultured for 24 h, fixed with 4% paraformaldehyde, and stained with 100 ng/ml DAPI (4′,6-diamidino-2-phenylindole) solution. The green and blue fluorescence signals were detected and merged.

### Preparation and Optimization of Double-Strand RNA

To better understand the function of *LvVEGFR2*, we detected the effects of interference of endogenous *LvVEGFR2* on the propagation of WSSV in *L. vannamei*. One pair of primers with T7 promoter sequences, LvVEGFR2-dsF and LvVEGFR2-dsR (Table 1) were designed to amplify a 701-bp cDNA fragments of *LvVEGFR2* as the template for dsRNA synthesis. Primers of dsEGFP-F and dsEGFP-R with T7 promoter sequences (Table 1) were used to amplify a 289-bp DNA fragment of EGFP gene as negative control. The PCR amplification program was as follows: denaturation at 94°C for 5 min; 35 cycles of denaturation at 94°C for 30 s, annealing at 59°C for 30 s and extension at 72°C for 30 s; final extension at 72°C for 10 min. The PCR products were purified using TIANgel Midi purification kit (Tiangen, China). The purified products were used to synthesize the corresponding dsRNAs using TranscriptAid T7 High Yield Transcription Kit (Thermo Fisher Scientific, USA). Redundant single-strand RNA was digested by RNaseA (Takara, Beijing). The concentration of synthesized dsRNA was assessed by Nanodrop 2000 (Thermo Fisher Scientific, USA).

In order to optimize the silencing efficiency of dsRNA of *LvVEGFR2*, 30 healthy shrimp were divided into six groups. Shrimp in each group were injected with negative control or dsRNA with different dosages, including 1, 2, and 4 µg for each individual. At 48 h after interference, cephalothoraxes of four individuals in each group were isolated and frozen in liquid nitrogen for total RNA extraction. The transcription level of *LvVEGFR2* was detected by qPCR with primers LvVEGFR2-qF and LvVEGFR2-qR as described in Section “Quantitative Real-time PCR Detection of LvVEGFR2 mRNA Expression.”

### RNA Interference and WSSV Infection

In order to explore the effect of *LvVEGFR2* silencing on WSSV propagation, 40 shrimp were divided into 2 groups including dsEGFP group and dsVR2 group. One microgram of dsRNA was injected into each shrimp after optimization of RNA interference effect. One microgram of dsEGFP and 1 µg dsLvVEGFR2 dissolved in 20-µl PBS was injected into each shrimp in dsEGFP group and dsVR2 group, respectively. At 48 h after dsRNA injection, 10^4^ copies of WSSV dissolved in 10 µl PBS was injected into each individual. The pleopods of 15 individuals from each group were collected at 24 and 36 h after WSSV injection. The samples collected from each group at each time were divided into three subgroups and frozen in liquid nitrogen for DNA extraction and WSSV copy number detection.

DNA was extracted from the frozen pleopods using Genomic DNA Kit (Tiangen, China) following the manufacturer’s instructions. The WSSV copy number in each DNA samples was quantified by qPCR with primers VP28-qF and VP28-qR (Table 1) according to the method described by Sun et al. (20). Briefly, the plasmid DNA containing a 281 bp fragment of *VP28* gene from WSSV was constructed and transfected into *Escherichia coli*. The plasmid was then extracted and quantified, and the copy number was calculated. Standard curve was constructed using 10-fold dilutions of the plasmid DNA ranging from 10^8^ to 10^3^. The qPCR was performed with the diluted plasmid DNA and the pleopods DNA under the following conditions: denaturation at 94°C for 2 min; 40 cycles of 94°C for 20 s, 55°C for 20 s, and 72°C for 20 s. The WSSV copy number per nanogram of pleopods DNA was calculated based on the standard curve.

### Analysis of the Effects of LvVEGFs on the Expression Regulation of LvVEGFR2

#### Detection on the Transcriptional Level of LvVEGFR2 after Silencing of LvVEGFs

Previously, we identified three VEGF genes in *L. vannamei* (17, 18). In order to explore the effect of *LvVEGF* genes on *LvVEGFR2*, the samples used in the previous studies, including shrimp at 48 h after RNA interference by dsRNA of LvVEGF1, LvVEGF2, and LvVEGF3 were used to detect expression of *LvVEGFR2*. QPCR with primers LvVEGFR2-qF and LvVEGFR2-qR (Table 1) were performed as described in Section “Quantitative Real-time PCR Detection of LvVEGFR2 mRNA Expression.”

#### Detection on the Transcription Level of LvVEGFR2 after Injection of Recombinant LvVEGFs

Primers LvVEGF1-pF/LvVEGF1-pR, LvVEGF2-pF/LvVEGF1-pR, and LvVEGF3-pF/LvVEGF3-pR were designed to amply the ORF excluding sequence encoding signal peptide of *LvVEGF1, LvVEGF2*, and *LvVEGF3*. The PCR fragments were cloned into the pCzn 1 vector (Zoonbio Biotechnology, China) to construct recombinant plasmids. The recombinant plasmid pCzn 1-VF1, pCzn 1-VF2, and pCzn 1-VF3 were transformed and successfully expressed in competent cells *E. coli* BL21 (DE3). Recombinant LvVEGFs protein pVF1, pVF2, and pVF3 were examined by SDS-polyacrylamide gel electrophoresis (SDS-PAGE), and purified by Ni-IDA-Sepharose CL-6B column (Bioz, USA). The purified pVF1, pVF2, and pVF3 protein were quantified and diluted to the concentration of 1.8 µg/µl.

Twenty experimental shrimp were divided into four groups. Each group was injected with 36 µg pVF1, pVF2, pVF3, or pET30A dissolved in 20 µl PBS, respectively. In addition, 20 µl PBS was injected into shrimp as blank group. At 3 h after injection of pVF1, pVF2, or pVF3 protein, cephalothoraxes of four individuals in each group were collected and frozen in liquid nitrogen for total RNA extraction and cDNA synthesis as described in Section “Total RNA Extraction and cDNA Synthesis.” The transcriptional levels of *LvVEGFR2* in these samples were detected through qPCR as described in Section “Quantitative Real-time PCR Detection of LvVEGFR2 mRNA Expression.”

### Yeast Two-Hybrid Assay

#### Plasmid Construction

The ORF excluding sequence encoding signal peptide of *LvVEGF1, LvVEGF2*, and *LvVEGF3* was amplified by primers LvVEGF1-pF/LvVEGF1-pR, LvVEGF2-pF/LvVEGF1-pR, and LvVEGF3-pF/LvVEGF3-pR, and then cloned into pGADT7 vector (Takara, Beijing), respectively. They were designated as pGAD-VF1, pGAD-VF2, and pGAD-VF3, and as prey plasmids. The extracellular region containing the first four IG domains of LvVEGFR2 [LvVR2(1–4)] was amplified by primers LvVEGFR2-pF/rLvVEGFR2-pR and then cloned into pGBKT7 vector (Takara, Beijing), which was designated as pGBK-VR2(1–4) and used as bait plasmid.

#### Yeast Transformation and Galactosidase Assays

The prey plasmid pGAD-VF1, pGAD-VF2, or pGAD-VF3 was co-transformed into yeast strain Y2H Gold with the bait plasmid pGBK-VR2(1–4) by the lithium acetate transformation procedure according to CLONTECH Matchmaker protocol manual (Clontech, USA). Transformants were then plated directly onto selective growth media, SD/-Leu/-Trp (DDO) plate. DDO is a commercial synthetic defined medium lacking both of leucine and tryptophan for selecting the bait and prey plasmids (Clontech, USA). Clones which contained both bait and prey plasmids could grew on the DDO plate. In addition, prey plasmids co-transformed with pGBKT7 and pGBK-VR2(1–4) co-transformed with pGADT7 were used to detect auto-activation. The co-transformed control plasmids pGBK-p53 and pGAD-T antigen were used as positive control, and pGBK-Lam and pGAD-T antigen were used as negative control, respectively. Transformants were allowed to grow at 30°C, usually for 3–5 days, until colonies were large enough (diameter 2–3 mm usually) for galactosidase activity assay.

After colonies grown, several fast-growing colonies were picked up and plated onto SD/-Leu/-Trp/X-α-Gal/AbA (DDO/X/A) plate for primary screening. The plates were allowed to grow at 30°C, usually for 3–5 days. After 3–5 days, colonies were then picked and plated onto SD/-Ade/-His/-Leu/-Trp/X-α-Gal/AbA (QDO/X/A) plate. QDO is also a commercial synthetic defined medium lacking Adenine, histidine, leucine, and tryptophan. X-α-Gal and Aureobasid in A were added into QDO to make up QDO/X/A. Yeast colonies that express *Mel1* turn blue in the presence of the chromogenic substrate X-α-Gal (Clontech, USA). This medium was used at the end of the two-hybrid screen to confirm protein interactions.

### Detection on the Expression Levels of LvFAK and LvPI3K

In order to know whether *LvVEGFR2* could regulate downstream signaling pathways, we detected the expression levels of *LvFAK* and *LvPI3K* after silencing of *LvVEGFR2* gene. Primers LvFAK-qF/LvFAK-qR and LvPI3K-qF/LvPI3K-qR (Table 1) were used to detect the expression level *LvFAK* and *LvPI3K* genes. The program was as follows: denaturation at 94°C for 2 min; 40 cycles of 94°C for 15 s, annealing temperature for 20 s, and 72°C for 20 s. The PCR product was denatured to produce melting-curve to check the specificity of the PCR product.

### Data Analysis

All the assays described above were biologically repeated for three times. For quantitative real-time PCR, four replicates were set for each sample. The relative transcription level of *LvVEGFR2* were obtained using 2^−ΔΔCt^ method (22) and the WSSV copy number per nanogram of DNA was obtained according to the standard curve. In addition, the numerical data from each experiment were analyzed to calculate the mean and standard deviation of triplicate assays. An independent sample *t*-test was used to analyze the difference between two groups by SPSS 16.0. *P* < 0.05 was considered statistically significant.

## Results

### The Full-Length cDNAs of LvVEGFR2 and Sequence Analysis

**Figure 1:**
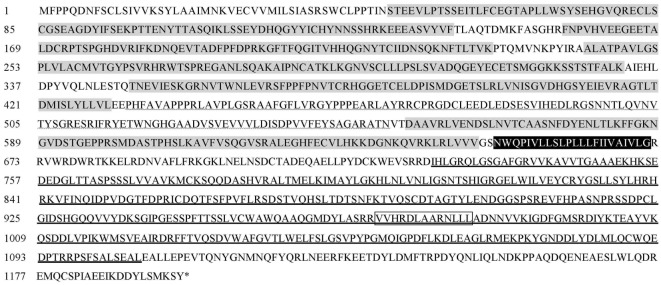
Deduced amino acid sequence of *LvVEGFR2* (GenBank accession: MF417824). Predicted domains were shown with different makers. Immunoglobulin subtype (IG) domains were marked in gray. Immunoglobulin-like domain was wave-underlined. Transmembrane motif (TM) domain was in dark. The protein kinase domain was bolded underlined and PK_TYR was bolded underlined and marked in box.

**Figure 2:**
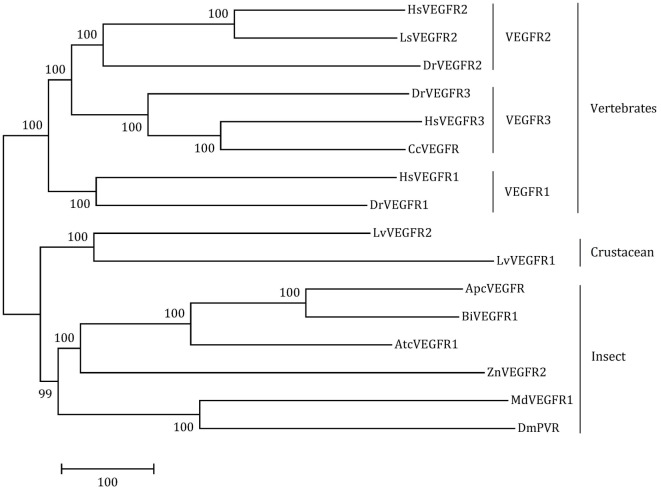
The neighbor-joining phylogenetic tree of LvVEGFR1, LvVEGFR2, and other homologous genes from other species (the information of VEGFRs is shown in Table 2).

The full-length ORF of *LvVEGFR2* cDNA was 3,594 bp encoding 1,197 amino acid residues (accession number: MF417824). As shown in Figure 1, the deduced amino acids sequence of LvVEGFR2 contained five immunoglobulin subtype domains (Ser^48^ to Phe^141^, Phe^156^ to Lys^230^, Ala^243^ to Lys^331^, Thr^349^ to Leu^430^, and Asp^555^ to Val^646^) and one immunoglobulin-like domain (Pro^413^ to Gln^552^) were predicted in the extracellular region. TM of 21 aa was located from Asn^649^ to Gly^671^ followed by a protein kinase (PK) domain from Ile^726^ to Leu^1007^. Moreover, a tyrosine-PK active site (Val^971^ to Leu^983^) existed in the PK domain. Phylogenetic analysis showed that LvVEGFR2 was first clustered together with LvVEGFR1 and then with VEGFR members from insects, while VEGFRs from vertebrates were clustered into another branch (Figure 2). Sequence alignment showed a 55% similarity between LvVEGFR1 and LvVEGFR2 amino acid sequences (data not shown).

### Tissue Distribution and Subcellular Localization of LvVEGFR2

**Figure 3:**
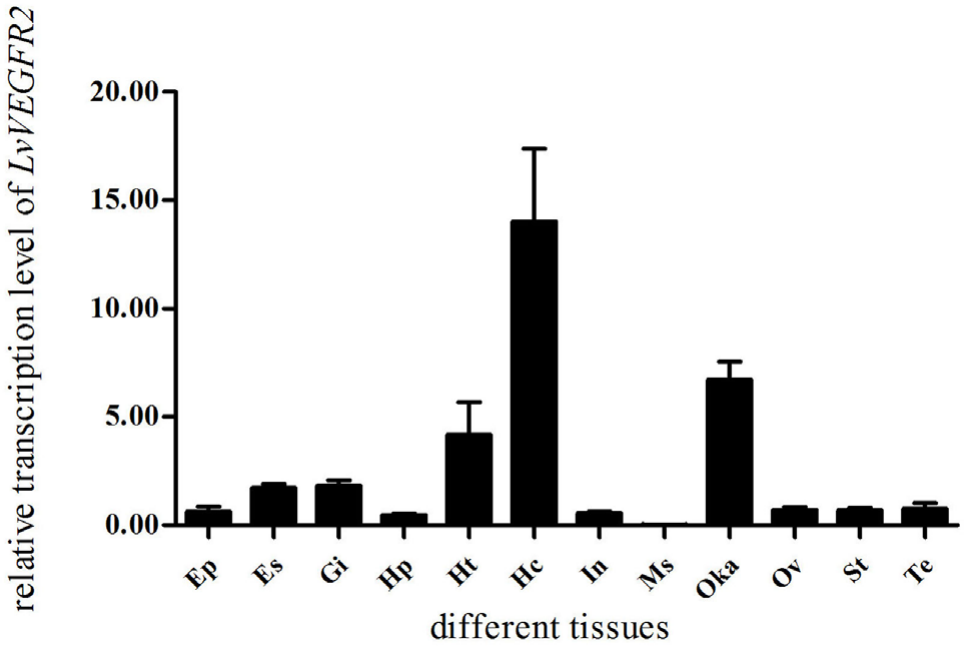
Distribution of *LvVEGFR2* transcripts in different tissues of *L. vannamei*. Ht, heart; Hp, hepatopancreas; Gi, gill; Oka, lymph organ; Ms, muscle; In, intestine; Hc, hemocytes; Es, eyestalk; St, stomach; Ep, epidermis; Te, testis; Ov, ovary.

The expression levels of *LvVEGFR2* in different tissues were shown in Figure 3. The transcript of *LvVEGFR2* was mainly detected in hemocytes, followed by Oka and heart. The expression level of *LvVEGFR2* was relatively lower in other tissues.

**Figure 4:**
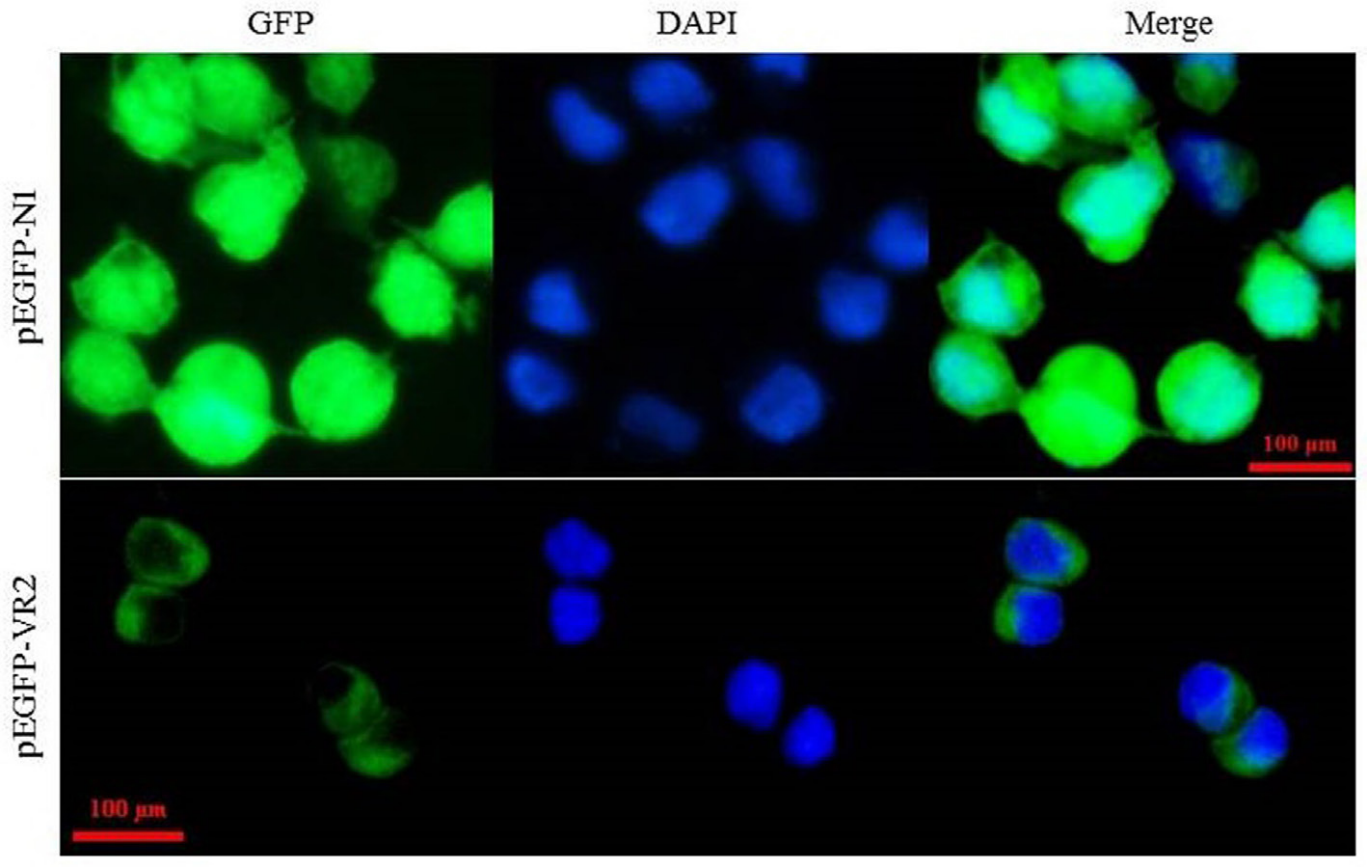
Subcellular localization of LvVEGFR2 in mammalian 293T cells. The plasmid pEGFP-VR2, which was constructed based on the plasmid pEGFP-N1, contained an N-terminal IgK_secretion tag, the full length of enhanced green fluorescent protein (EGFP), and a C-terminal amino acid sequence including the predicted TM of LvVEGFR2 and its flanking sequence. The plasmid pEGFP-VR2 and control plasmid pEGFP-N1 were transfected into 293T cells and the fluorescence signals were detected, respectively. GFP showed fluorescence signals generated by expressed EGFP protein. DAPI showed signals generated by stained nucleus. Merge showed the merged signals of GFP and DAPI.

Subcellular localization analysis showed that the fluorescence signals expressed by the constructed plasmid pEGFP-VR2 that contained the predicted transmembrane motif of LvVEGFR2 were mainly detected on the cell membrane of 293T cells, while the fluorescence signals expressed the control plasmid pEGFP-N1 were mainly detected in the cytoplasm of 293T cells (Figure 4).

### Expression Profile of LvVEGFR2 in Shrimp after WSSV Challenge

**Figure 5:**
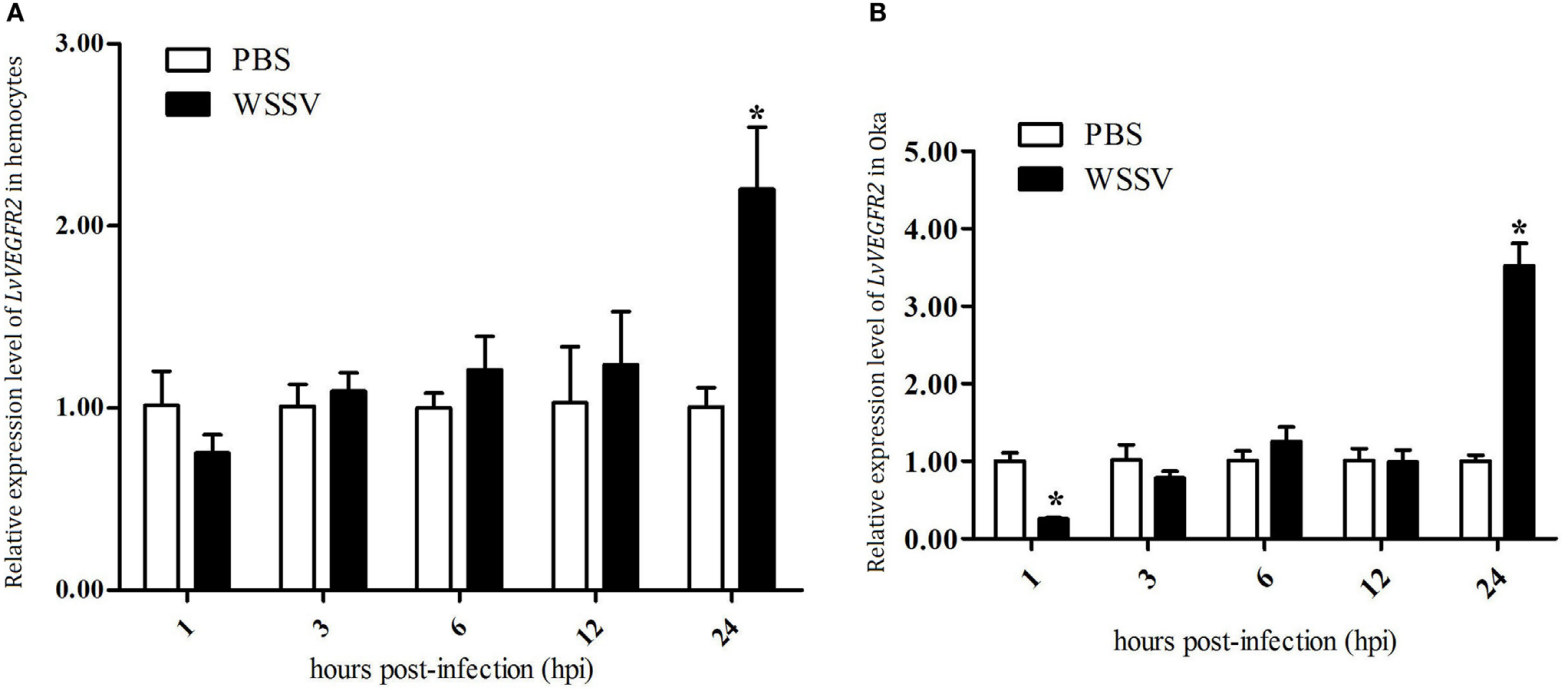
Expression profile of *LvVEGFR2* in hemocytes **(A)** and Oka **(B)** at different time post WSSV infection (hpi). PBS, phosphate-buffered saline injection group; WSSV, white spot syndrome virus injection group. Stars (*) indicate significant differences (*P* < 0.05) for the gene expression levels between PBS and WSSV groups.

The expression profiles of *LvVEGFR2* in hemocytes and Oka of shrimp after WSSV challenge were detected. The expression levels of *LvVEGFR2* in hemocytes (Figure 5A) and Oka (Figure 5B) were significantly upregulated at 24 hour post WSSV infection (hpi).

### Silencing of LvVEGFR2 Affected *In Vivo* Virus Propagation

**Figure 6:**
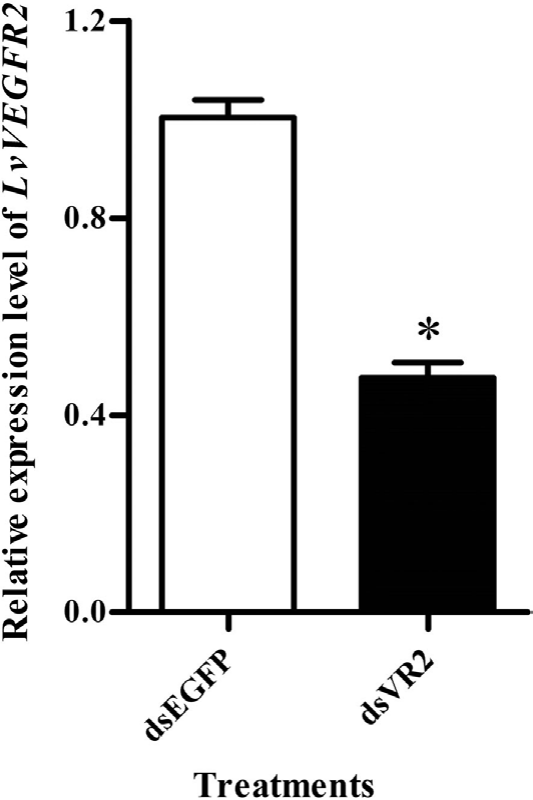
Expression level of *LvVEGFR2* in the cephalothoraxes of shrimp after injection 48 h of dsRNAs. dsEGFP, injected with dsEGFP; dsVR2, injected with dsRNA designed for *LvVEGFR2*. Stars (*) show significant differences (*P* < 0.05) for the gene expression levels between two groups.

RNA interference based on dsRNA was performed to study the function of *LvVEGFR2* gene. First, the silencing efficiency of *LvVEGFR2* was detected under different dsRNA dosages, including 1, 2, and 4 µg for each shrimp. After optimization to the dsRNA dosage for interference, the dose of 4 µg dsRNA per shrimp, with 52.5% inhibition efficiency (Figure 6), was used for further RNAi experiment.

**Figure 7:**
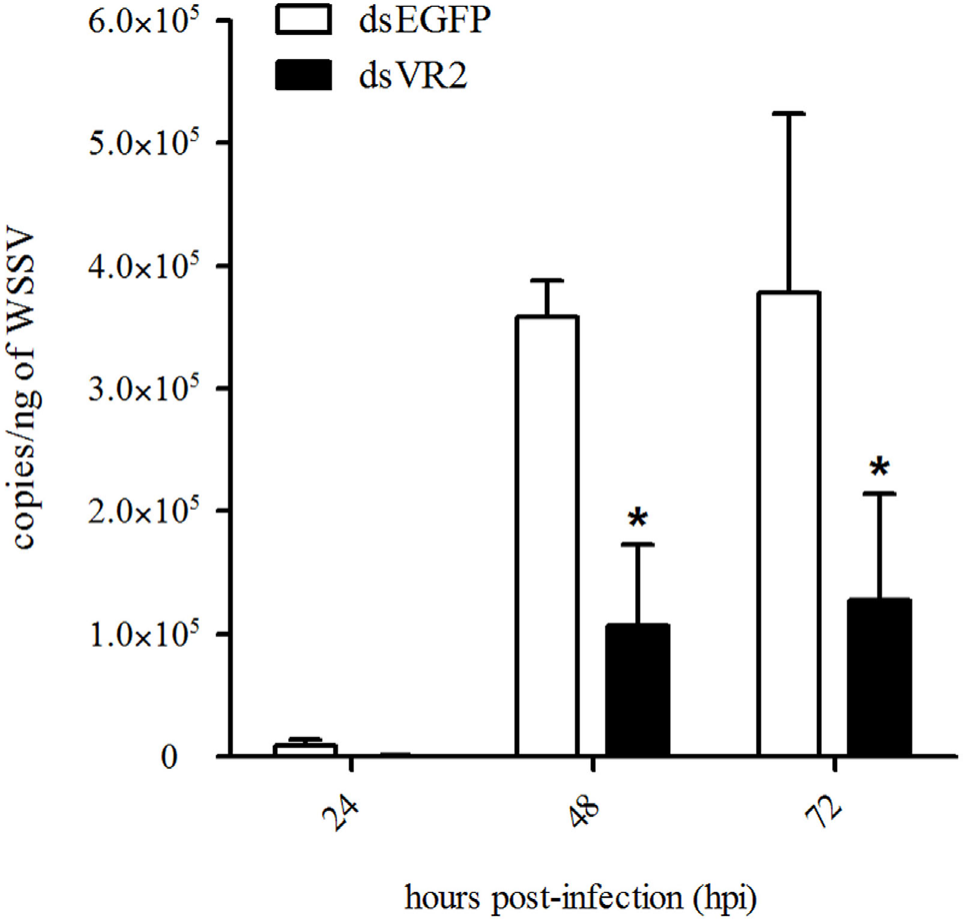
Amount of white spot syndrome virus (WSSV) particles in shrimp at different time (hours, h) after silencing of LvVEGFR2 and WSSV infection. dsEGFP, injected with dsEGFP and WSSV; dsVR2, injected with dsLvVEGFR2 and WSSV. Stars (*) show significant differences (*P* < 0.05) of the gene expression levels under different treatments.

The WSSV copy number was detected in shrimp after dsRNA and WSSV injection. At 48 and 72 hpi, the WSSV copy number in shrimp from dsEGFP group was about 4 × 10^5^ copies/ng DNA, while it was about 1 × 10^5^ copies/ng DNA in shrimp from dsVR2 group, which was significantly lower (*P* < 0.05) than that of dsEGFP group (Figure 7).

### Silencing of LvVEGFs Reduced the Transcription Level of LvVEGFR2

**Figure 8:**
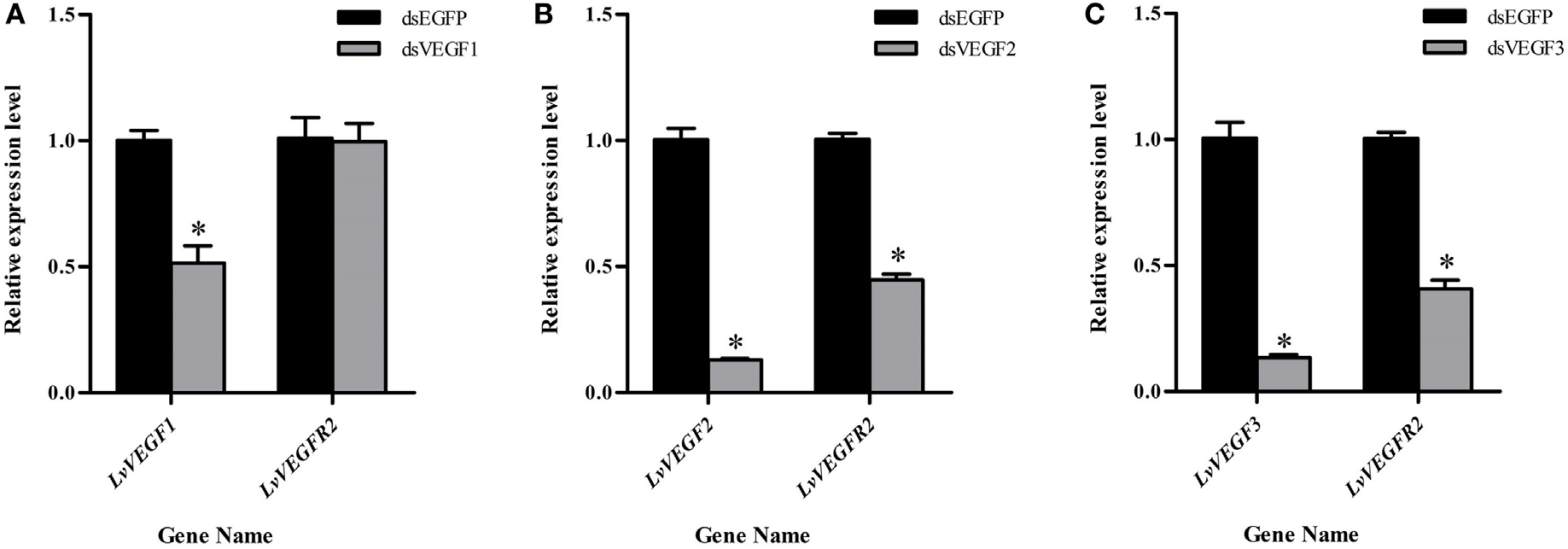
Expression level of *LvVEGFR2* in the cephalothorax of shrimp after injection of dsVF1 **(A)**, dsVF2 **(B)**, and dsVF3 **(C)**. dsEGFP, injected with dsEGFP; dsVF1, injected with dsLvVEGF1; dsVF2, injected with dsLvVEGF2; dsVF3, injected with dsLvVEGF2. Stars (*) show significant differences (*P* < 0.05) for the gene expression levels between two groups.

In order to know whether *LvVEGFs* could regulate the transcription of *LvVEGFR2, LvVEGF1, LvVEGF2*, and *LvVEGF3* were silenced by dsRNA and then the transcriptional level of *LvVEGFR2* was detected. After silencing of *LvVEGF1* by 48.6%, the transcriptional level of *LvVEGFR2* had no obvious change (Figure 8A). After silencing of *LvVEGF2* by 87.0%, the transcriptional level of *LvVEGFR2* was downregulated by 55.3% (Figure 8B). After silencing of *LvVEGF3* by 86.6%, the transcriptional level of *LvVEGFR2* was downregulated by 59.3% (Figure 8C).

### Injection of Recombinant LvVEGFs Protein Upregulated the Transcription Level of LvVEGFR2

**Figure 9:**
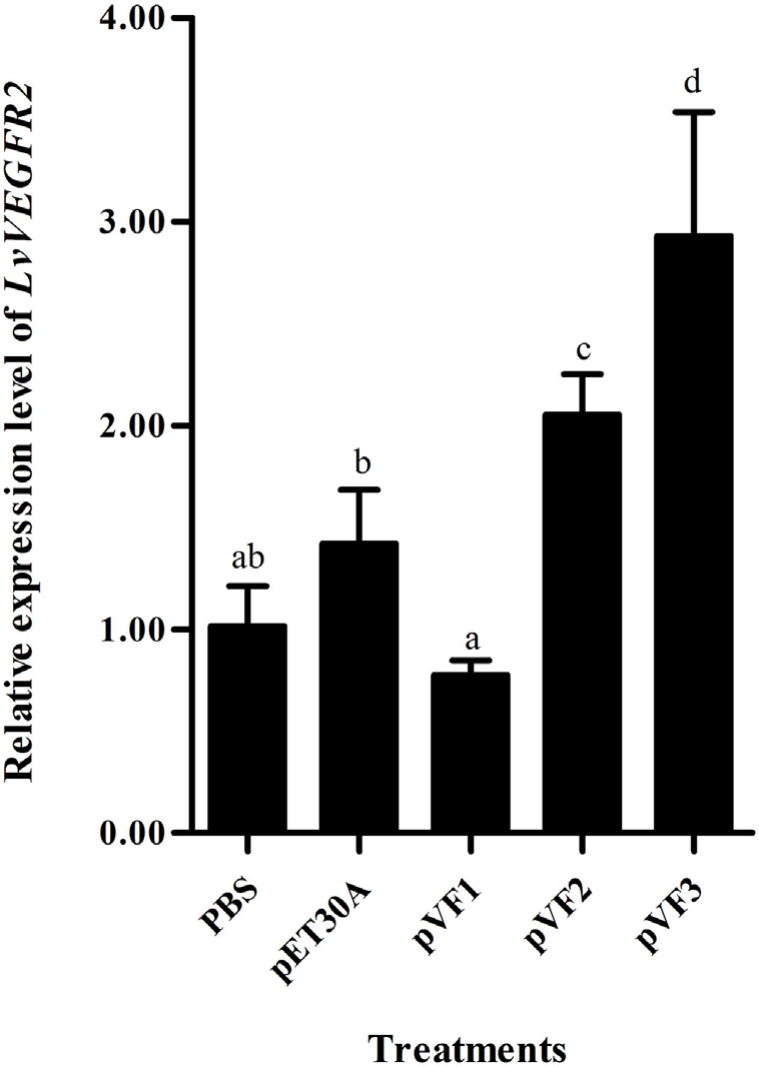
Expression level of *LvVEGFR2* in the cephalothorax of shrimp after injection of pVF1, pVF2, and pVF3. PBS, injected with phosphate-buffered saline; pET30A, injected with pET30A protein; pVF1, injected with recombinant LvVEGF1 protein; pVF2, injected with recombinant LvVEGF2 protein; pVF3, injected with recombinant LvVEGF3 protein. Different lowercase letters (a, b, c, and d) show significant differences (*P* < 0.05) for the gene expression levels between two groups.

Injection of the recombinant protein pVF1, pVF2, and pVF3 could also influence the transcriptional level of *LvVEGFR2*. As shown in Figure 9, injection of pVF1 did not upregulate the transcriptional level of *LvVEGFR2* when compared to pET30A injection and PBS control. After injection of pVF2 or pVF3, the transcriptional level of *LvVEGFR2* was obviously upregulated by 44 and 106% compared to pET30A injection and by 104 and 192% when compared with PBS control, respectively.

### Detection on the Interaction between LvVEGFs and LvVEGFR2 by Yeast Two-Hybrid System

**Figure 10:**
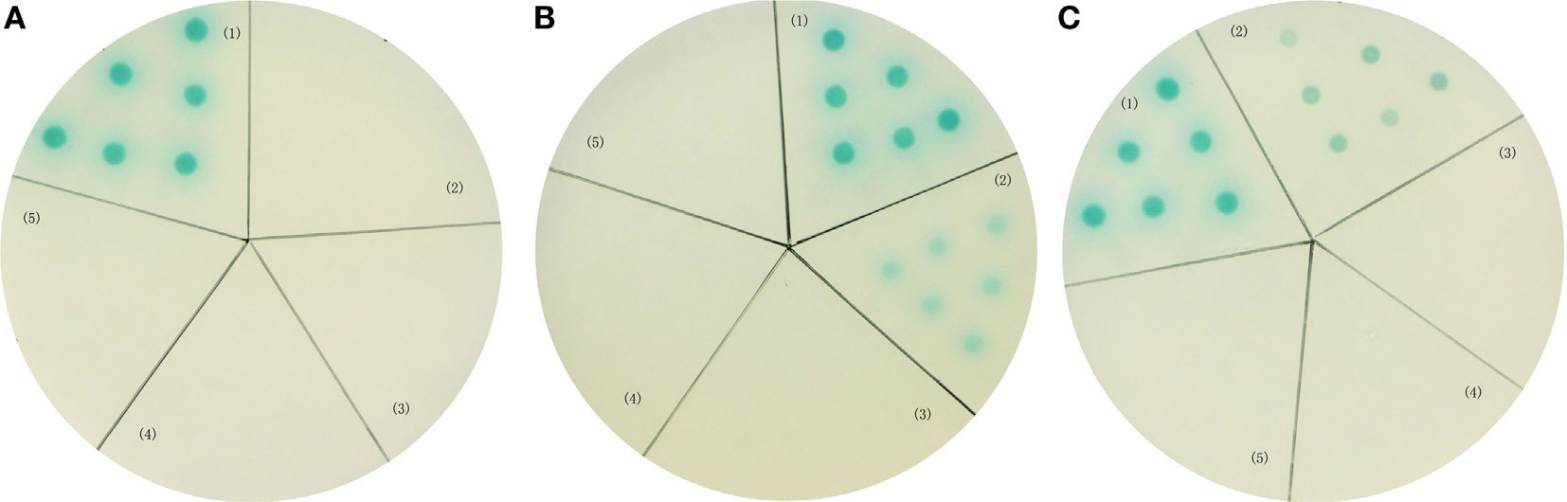
Yeast two-hybrid analysis. Yeast cells were transformed with a combination of the indicated plasmids. **(A)** (1) pGBK-p53 and pGAD-T antigen for positive control; (2) pGAD-VF1 and pGBK-VR2(1–4) plasmids; (3) pGAD-VF1 and pGBKT7 plasmids; (4) pGBK-VR2(1–4) and pGADT7 plasmids; (5) pGBK-Lam and pGAD-T antigen for negative control. **(B)** (1) pGBK-p53 and pGAD-T antigen for positive control; (2) pGAD-VF2 and pGBK-VR2(1–4) plasmids; (3) pGAD-VF2 and pGBKT7 plasmids; (4) pGBK-VR2(1–4) and pGADT7 plasmids; (5) pGBK-Lam and pGAD-T antigen for negative control. **(C)** (1) pGBK-p53 and pGAD-T antigen for positive control; (2) pGAD-VF3 and pGBK-VR2(1–4) plasmids; (3) pGAD-VF3 and pGBKT7 plasmids; (4) pGBK-VR2(1–4) and pGADT7 plasmids; (5) pGBK-Lam and pGAD-T antigen for negative control.

In order to further investigate the interaction between LvVEGFs and LvVEGFR2, the yeast two-hybrid system was used. Expression of reporter genes in the positive control, which was co-transformed with pGBK-p53 and pGAD-T antigen plasmids, could generate blue color in the yeast cells (Figures 10A–C, zone 1). When pGAD-VF2 and pGBK-VR2(1–4) plasmids or pGAD-VF3 and pGBK-VR2(1–4) plasmids were co-expressed in the yeast cells, the blue color was also detected (Figures 10B,C, zone 2), while co-transformation of pGAD-VF1 and pGBK-VR2(1–4) plasmids into the yeast cells did not generate blue color (Figure 10A, zone 2). These data indicated that LvVEGF2 and LvVEGF3 could interact with the Ig domains region of LvVEGFR2. Self-activation and negative controls were set and no blue signal was detected (Figures 10A–C, zones 3–5).

### Silencing of LvVEGFR2 Decreased Expression Levels of LvFAK and LvPI3K Genes

**Figure 11:**
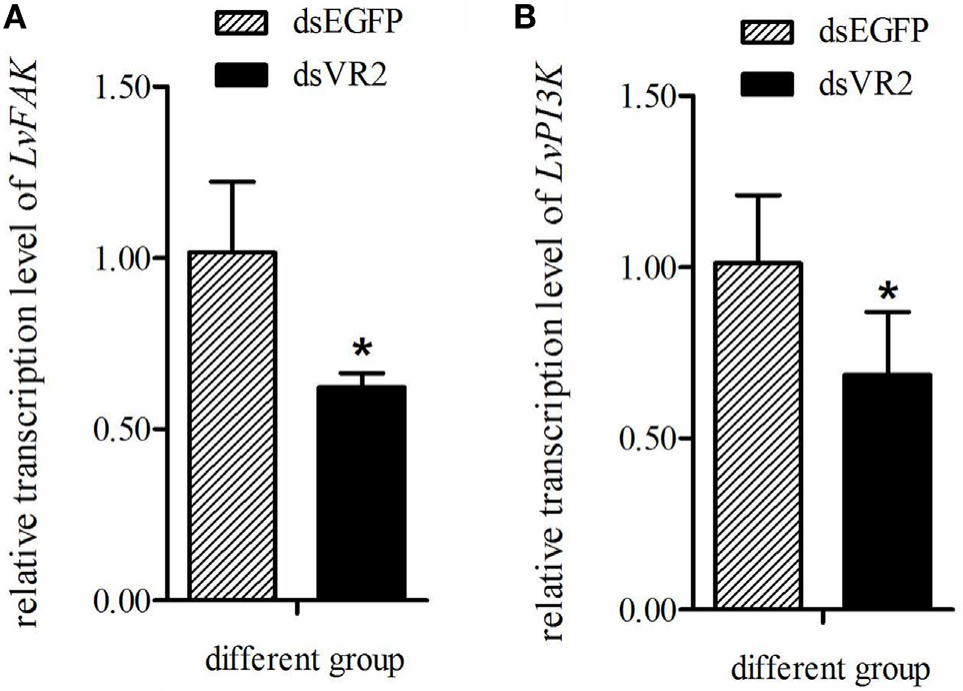
Expression level of *LvFAK* **(A)** and *LvPI3K* **(B)** in the cephalothoraxes of shrimp after silencing of *LvVEGFR2*. dsEGFP, injected with dsEGFP; dsVR2, injected with dsLvVEGFR2. Stars (*) show significant differences (*P* < 0.05) for the gene expression level between two groups.

In order to know whether *LvVEGFR2* had impact on other signaling pathways, we detected the expression levels of *LvFAK* and *LvPI3K* in shrimp after *LvVEGFR2* silencing. After silencing of *LvVEGFR2* gene, the expression level of *LvFAK* and *LvPI3K* was downregulated by about 38 and 32%, respectively (Figure 11).

## Discussion

The VEGF signaling pathway in mammals is considered essential in many physiological processes in angiogenesis, hematopoiesis and lymphogenesis (23, 24). In crustacean, VEGF signaling pathway participates in innate immunity and is responsive to pathogen infection (13, 14). Previously, we characterized a *VEGFR* gene (*LvVEGFR1*) and three *VEGF* genes in *L. vannamei* and data showed that they were all involved in WSSV infection (16–18). Here, we identified a new VEGFR gene, *LvVEGFR2*, in *L. vannamei* and characterized its function during WSSV infection.

VEGFR is a kind of tyrosine kinase receptor which carries several immunoglobulin-like domains, a single transmembrane domain, a juxtamembrane domain, and a kinase domain split by a kinase insert and a carboxyl terminus (24). Sequence analysis showed that LvVEGFR2 shared similar intracellular domains with VEGFR family members. In the extracellular region, most VEGFRs such as LvVEGFR1, HsVEGFR1, HsVEGFR2, and DmVEGF-R had seven IG-like domains, while the extracellular region of LvVEGFR2 was composed of six IG-like domains which were similar to HsVEGFR3 and *DmVEGFR* (1, 2, 25). Although phylogenetic analysis showed that LvVEGFR2 was first clustered with LvVEGFR1, the sequence similarity between them was only 55%. These data indicated that LvVEGFR2 was a novel VEGFR gene which was distinguished from LvVEGFR1 in *L*. *vannamei*.

In order to know whether *LvVEGFR2* was involved in immune response in shrimp, we first detected its spatial distribution and its temporal expression during WSSV infection. *LvVEGFR2* showed the highest expression levels in hemocytes and Oka. Hemocytes play key roles in crustacean innate immunity such as phagocytosis (26, 27), storage and release of the proPO (28), and so on. Oka is one of the most specific and effective organ for clearance of bacteria (29) and virus (30). At 24 h post WSSV infection, the expression level of *LvVEGFR2* gene in hemocytes and Oka was significantly upregulated. Different from *LvVEGFR2*, the previously reported *LvVEGFR1* gene was upregulated at 1 h post WSSV infection in hemocytes (16). The data indicated that *LvVEGFR1* might be an early response gene to WSSV infection, while *LvVEGFR2* might be a late response gene. It should be also noted that there was a significant decrease of *LvVEGFR2* expression at 1 hpi in Oka, which was similar to the decrease of *LvVEGFR1* at 1 hpi in intestine (16). A possible reason for the decreases might be that WSSV infection recruited VEGFs from other tissues into hemolymph since WSSV would first enter the hemolymph after muscle injection.

Interference of *LvVEGFR1* gene by double-strand RNA could inhibit the *in vivo* propagation of WSSV in shrimp (16). Silencing of other members in shrimp VEGF signaling pathway, including *LvVEGF1, LvVEGF2*, and *LvVEGF3* genes, could also reduce WSSV copy number in WSSV-infected shrimp (17, 18). Similarly, silencing of *LvVEGFR2* could obviously reduce the virus copy number in WSSV-infected shrimp. These data collectively supported the previous conclusion that inhibition of the VEGF signaling pathway could restrict the propagation of WSSV in shrimp.

The VEGF signaling pathway is directly activated by binding VEGFs to their receptors VEGFRs. The extracellular IG domains of VEGFRs are considered as the binding site of VEGFs (3, 4). In shrimp *L*. *vannamei*, silencing of *LvVEGF1* and *LvVEGF2* genes with dsRNA reduced the expression level of *LvVEGFR1* gene (17), and LvVEGF3 could interact with the second to fifth Ig domains of LvVEGFR1 under yeast two-hybrid system (18), which indicated that LvVEGFR1 might be the receptor for LvVEGF1, LvVEGF2, and LvVEGF3. In the present study, the transcription level of *LvVEGFR2* was downregulated after silencing of *LvVEGF2* and *LvVEGF3*, and upregulated by injection of recombinant LvVEGF2 and LvVEGF3 proteins. However, silencing of *LvVEGF1* and injection of recombinant LvVEGF1 did not influence the expression level of *LvVEGFR2* gene. Meanwhile, LvVEGF2 and LvVEGF3, rather than LvVEGF1, could interact with the extracellular Ig domains of LvVEGFR2. These data indicated that the LvVEGFR2 was also the receptor of LvVEGF2 and LvVEGF3.

After activation, VEGFRs usually function through regulating many genes in cellular signaling pathways such as STAT (31), PI3K/Akt (32), FAK (33), ERK (34), and so on. These genes or related signaling pathways play important roles during WSSV infection in crustaceans. WSSV infection could activate STAT (35) and then use it to promote WSSV *ie1* gene expression (36). FAK could not only promote WSSV infection but also participate in immune defense (37). Suppression of PI3K–Akt–mTOR pathway inhibited *in vivo* WSSV propagation (38). Presently, the expression level of *LvFAK* and *LvPI3K* was downregulated after *LvVEGFR2* silencing, which was similar to our previous reports on the *LvVEGFR1* and *LvVEGFs* genes (16–18). Therefore, we inferred that the VEGF signaling pathway in shrimp affected WSSV propagation might through regulating downstream signaling pathways.

In conclusion, a novel *VEGFR* gene (*LvVEGFR2*) was identified in shrimp *L. vannamei* in the present study. *LvVEGFR2* was responsive to WSSV infection and could regulate the *in vivo* propagation of WSSV. LvVEGFR2 interacted with extracellular ligands LvVEGF2 and LvVEGF3, and they could regulate expression of downstream genes including *LvFAK* and *LvPI3K*. These results, together with our previous report on *LvVEGFR1* and *LvVEGFs*, collectively indicated that the VEGF signaling pathway in shrimp might participate in WSSV infection through regulating its downstream signaling pathways.

## Author Contributions

SL, FL, and JX conceived and designed the project. SL and ZW performed all the experiments and prepared the figures. KY prepared for the experimental animals. SL, ZW, and FL wrote the manuscript. All authors reviewed the manuscript.

## Conflict of Interest Statement

The authors declare that the research was conducted in the absence of any commercial or financial relationships that could be construed as a potential conflict of interest.

## Footnotes

^1^http://www.ncbi.nlm.nih.gov/gorf/gorf.html.

^2^http://www.ncbi.nlm.nih.gov/blast.

^3^http://www.ebi.ac.uk/interpro/.

^4^http://smart.embl-heidelberg.de/.

^5^http://www.ebi.ac.uk/Tools/msa/clustalw2/.

## References

[1] Tammela T Enholm B Alitalo K Paavonen K. The biology of vascular endothelial growth factors. Cardiovasc Res (2005) 65:550–63. 10.1016/j.cardiores.2004.12.002 15664381

[2] Shibuya M. Vascular endothelial growth factor (VEGF) and its receptor (VEGFR) signaling in angiogenesis: a crucial target for anti- and pro-angiogenic therapies. Genes Cancer (2011) 2(12):1097–105. 10.1177/1947601911423031 22866201 PMC3411125

[3] Christinger HW Fuh G de Vos AM Wiesmann C. The crystal structure of placental growth factor in complex with domain 2 of vascular endothelial growth factor receptor-1. J Biol Chem (2004) 279:10382–8. 10.1074/jbc.M313237200 14684734

[4] Fuh G Li B Crowley C Cunningham B Wells JA. Requirements for binding and signaling of the kinase domain receptor for vascular endothelial growth factor. J Biol Chem (1998) 273:11197–204. 10.1074/jbc.273.18.11197 9556609

[5] Bates DO Hillman NJ Williams B Neal CR Pocock TM. Regulation of microvascular permeability by vascular endothelial growth factors. J Anat (2002) 200(6):581–97. 10.1046/j.1469-7580.2002.00066.x 12162726 PMC1570751

[6] Nash AD Baca M Wright C Scotney PD The biology of vascular endothelial growth factor-B (VEGF-B). Pulm Pharmacol Ther (2006) 19(1):61–9. 10.1016/j.pupt.2005.02.007 16286239

[7] Stacker SA Caesar C Baldwin ME Thornton GE Williams RA Prevo R VEGF-D promotes the metastatic spread of tumor cells via the lymphatics. Nat Med (2001) 7(2):186–91. 10.1038/84635 11175849

[8] Joukov V Pajusola K Kaipainen A Chilov D Lahtinen I Kukk E A novel vascular endothelial growth factor, VEGF-C, is a ligand for the Flt4 (VEGFR-3) and KDR (VEGFR-2) receptor tyrosine kinases. EMBO J (1996) 15(2):290–8. 8617204 PMC449944

[9] Kukk E Lymboussaki A Taira S Kaipainen A Jeltsch M Joukov V VEGF-C receptor binding and pattern of expression with VEGFR-3 suggests a role in lymphatic vascular development. Development (1996) 122(12):3829–37. 9012504 10.1242/dev.122.12.3829

[10] Inder MK Ueda N Mercer AA Fleming SB Wise LM. Bovine papular stomatitis virus encodes a functionally distinct VEGF that binds both VEGFR-1 and VEGFR-2. J Gen Virol (2007) 88:781–91. 10.1099/vir.0.82582-0 17325350

[11] Meyer M Clauss M Lepple-Wienhues A Waltenberger J Augustin HG Ziche M A novel vascular endothelial growth factor encoded by Orf virus, VEGF-E, mediates angiogenesis via signalling through VEGFR-2 (KDR) but not VEGFR-1 (Flt-1) receptor tyrosine kinases. EMBO J (1999) 18:363–74. 10.1093/emboj/18.2.363 9889193 PMC1171131

[12] Wise LM Veikkola T Mercer AA Savory LJ Fleming SB Caesar C Vascular endothelial growth factor (VEGF)-like protein from Orf virus NZ2 binds to VEGFR2 and neuropilin-1. Proc Natl Acad Sci U S A (1999) 96:3071–6. 10.1073/pnas.96.6.3071 10077638 PMC15896

[13] Li F Xu L Gai X Zhou Z Wang L Zhang H The involvement of PDGF/VEGF related factor in regulation of immune and neuroendocrine in Chinese mitten crab *Eriocheir sinensis*. Fish Shellfish Immunol (2013) 35:1240–8. 10.1016/j.fsi.2013.07.042 23933264

[14] Inada M Yui T Kono T Yoshida T Sakai M Itami T Novel cytokine genes from Kuruma shrimp *Marsupenaeus japonicus*: MIF and VEGF are important in the innate immunity. Fish Shellfish Immunol (2013) 34:1656–7. 10.1016/j.fsi.2013.03.070

[15] Li SH Zhang XJ Sun Z Li FH Xiang JH. Transcriptome analysis on Chinese shrimp *Fenneropenaeus chinensis* during WSSV acute infection. PLoS One (2013) 8(3):e58627. 10.1371/journal.pone.0058627 23527000 PMC3602427

[16] Li SH Wang ZW Li FH Xiang JH. One type of VEGFR is involved in WSSV infection to the Pacific whiteleg shrimp *Litopenaeus vannamei*. Dev Comp Immunol (2015) 50:1–8. 10.1016/j.dci.2015.01.001 25576099

[17] Wang Z Li S Li F Yang H Yang F Xiang J. Characterization of two types of vascular endothelial growth factor from *Litopenaeus vannamei* and their involvements during WSSV infection. Fish Shellfish Immunol (2015) 47:824–32. 10.1016/j.fsi.2015.10.026 26492995

[18] Wang Z Li S Li F Xie S Xiang J. Identification and function analysis of a novel vascular endothelial growth factor, LvVEGF3, in the Pacific whiteleg shrimp *Litopenaeus vannamei*. Dev Comp Immunol (2016) 63:111–20. 10.1016/j.dci.2016.05.020 27241034

[19] Rodriguez J Boulo V Mialhe E Bachere E. Characterization of shrimp hemocytes and plasma components by monoclonal-antibodies. J Cell Sci (1995) 108:1043–50. 7622592 10.1242/jcs.108.3.1043

[20] Sun YM Li FH Xiang JH Analysis on the dynamic changes of the amount of WSSV in Chinese shrimp *Fenneropenaeus chinensis* during infection. Aquaculture (2013) 376:124–32. 10.1016/j.aquaculture.2012.11.014

[21] Wei J Zhang X Yu Y Huang H Li F Xiang J. Comparative transcriptomic characterization of the early development in Pacific white shrimp *Litopenaeus vannamei*. PLoS One (2014) 9(9):e106201. 10.1371/journal.pone.0106201 25197823 PMC4157780

[22] Livak KJ Schmittgen TD Analysis of relative gene expression data using real-time quantitative PCR and the 2^−ΔΔCT^ method. Methods (2001) 25:402–8. 10.1006/meth.2001.1262 11846609

[23] Jussila L Alitalo K. Vascular growth factors and lymphangiogenesis. Physiol Rev (2002) 82:673–700. 10.1152/physrev.00005.2002 12087132

[24] Rahimi N VEGFR-1 and VEGFR-2: two non-identical twins with a unique physiognomy. Front Biosci (2006) 11:818–29. 10.2741/1839 16146773 PMC1360224

[25] Parsons B Foley E. The *Drosophila* platelet-derived growth factor and vascular endothelial growth factor-receptor related (Pvr) protein ligands Pvf2 and Pvf3 control hemocyte viability and invasive migration. J Biol Chem (2013) 288:20173–83. 10.1074/jbc.M113.483818 23737520 PMC3711285

[26] Smith VJ Söderhäll K. Induction of degranulation and lysis of haemocytes in the freshwater crayfish, *Astacus astacus* by components of the prophenoloxidase activating system *in vitro*. Cell Tissue Res (1983) 233:295–303. 10.1007/BF00238297 6413069

[27] Söderhäll K Smith VJ Johansson MW Exocytosis and uptake of bacteria by isolated haemocyte populations of two crustaceans: evidence for cellular co-operation in the defence reactions of arthropods. Cell Tissue Res (1986) 245:43–9. 10.1007/BF00218085

[28] Johansson MW Söderhäll K Cellular immunity in crustaceans and the proPO system. Parasitol Today (1989) 5:171–6. 10.1016/0169-4758(89)90139-7 15463205

[29] Van de Braak CBT Botterblom MHA Taverne N Van Muiswinkel WB Rombout JHWM Van der Knaap WPW. The roles of haemocytes and the lymphoid organ in the clearance of injected *Vibrio* bacteria in *Penaeus monodon* shrimp. Fish Shellfish Immunol (2002) 13:293–309. 10.1006/fsim.2002.0409 12443012

[30] Hasson KW Lightner DV Mohney LL Redman RM White BM Role of lymphoid organ spheroids in chronic Taura syndrome virus (TSV) infections in *Penaeus vannamei*. Dis Aquat Organ (1999) 38:93–105. 10.3354/dao038093

[31] Chen H Ye D Xie X Chen B Lu W VEGF, VEGFRs expressions and activated STATs in ovarian epithelial carcinoma. Gynecol Oncol (2004) 94:630–5. 10.1016/j.ygyno.2004.05.056 15350351

[32] Abid MR Guo S Minami T Spokes KC Ueki K Skurk C Vascular endothelial growth factor activates PI3K/Akt/forkhead signaling in endothelial cells. Arterioscler Thromb Vasc Biol (2004) 24(2):294–300. 10.1161/01.ATV.0000110502.10593.06 14656735

[33] Chen XL Nam JO Jean C Lawson C Walsh CT Goka E VEGF induced vascular permeability is mediated by FAK. Dev Cell (2012) 22(1):146–57. 10.1016/j.devcel.2011.11.002 22264731 PMC3266538

[34] Li W Man XY Li CM Chen JQ Zhou J Cai SQ VEGF induces proliferation of human hair follicle dermal papilla cells through VEGFR-2-mediated activation of ERK. Exp Cell Res (2012) 318:1633–40. 10.1016/j.yexcr.2012.05.003 22659165

[35] Chen WY Ho KC Leu JH Liu KF Wang HC Kou GH WSSV infection activates STAT in shrimp. Dev Comp Immunol (2008) 32:1142–50. 10.1016/j.dci.2008.03.003 18460415

[36] Liu WJ Chang YS Wang AHJ Kou GH Lo CF. White spot syndrome virus annexes a shrimp STAT to enhance expression of the immediate-early gene *ie1*. J Virol (2007) 81:1461–71. 10.1128/JVI.01880-06 17079306 PMC1797513

[37] Zhang M Wang H Li D Xu X. A novel focal adhesion kinase from *Marsupenaeus japonicus* and its response to WSSV infection. Dev Comp Immunol (2009) 33(4):533–9. 10.1016/j.dci.2008.10.007 19013481

[38] Su MA Huang YT Chen IT Lee DY Hsieh YC Li CY An invertebrate warburg effect: a shrimp virus achieves successful replication by altering the host metabolome via the PI3K-Akt-mTOR pathway. PLoS Pathog (2014) 10(6):e1004196. 10.1371/journal.ppat.1004196 24945378 PMC4055789

